# Variants in Notch signalling pathway genes, *PSEN1* and *MAML2,* predict overall survival in Chinese patients with epithelial ovarian cancer

**DOI:** 10.1111/jcmm.13764

**Published:** 2018-07-28

**Authors:** Yuan Xu, Lei Cheng, Hongji Dai, Ruoxin Zhang, Mengyun Wang, Tingyan Shi, Menghong Sun, Xi Cheng, Qingyi Wei

**Affiliations:** ^1^ Cancer Institute Fudan University Shanghai Cancer Center Shanghai China; ^2^ Department of Oncology Shanghai Medical College Fudan University Shanghai China; ^3^ Department of Epidemiology and Biostatistics Key Laboratory of Cancer Prevention and Therapy Tianjin China; ^4^ Key Laboratory of Breast Cancer Prevention and Therapy Ministry of Education National Clinical Research Center for Cancer Tianjin Medical University Cancer Institute and Hospital Tianjin China; ^5^ Ovarian Cancer Program Division of Gynecologic Oncology Department of Gynecology and Obstetrics Fudan University Zhongshan Hospital Shanghai China; ^6^ Department of Pathology Tissue Bank Fudan University Shanghai Cancer Center Shanghai China; ^7^ Department of Gynecologic Oncology Fudan University Shanghai Cancer Center Shanghai China; ^8^ Duke Cancer Institute Duke University Medical Center Durham NC USA; ^9^ Department of Population Health Sciences Duke University School of Medicine Durham NC USA

**Keywords:** epithelial ovarian cancer, *MAML2*, Notch pathway, *PSEN1*, single nucleotide polymorphisms

## Abstract

To identify genetic variants in Notch signalling pathway genes that may predict survival of Han Chinese patients with epithelial ovarian cancer (EOC), we analysed a total of 1273 single nucleotide polymorphisms (SNPs) within 75 Notch genes in 480 patients from a published EOC genomewide association study (GWAS). We found that *PSEN1* rs165934 and *MAML2* rs76032516 were associated with overall survival (OS) of patients by multivariate Cox proportional hazards regression analysis. Specifically, the *PSEN1* rs165934 AA genotype was associated with a poorer survival (adjusted hazards ratio [adjHR] = 1.41, 95% CI = 1.07‐1.84, and *P* = .014), compared with the CC + CA genotype, while *MAML2* rs76032516 AA + AC genotypes were associated with a poorer survival (adjHR = 1.58, 95% CI = 1.16‐2.14, *P* = .004), compared with the CC genotype. The combined analysis of these two SNPs revealed that the death risk increased as the number of unfavourable genotypes increased in a dose‐dependent manner (*P*_trend_ < .001). Additionally, the expression quantitative trait loci analysis revealed that the SNP rs165932 in the rs165934 LD block (*r* ^2^ = .946) was associated with expression levels of *PSEN1*, which might be responsible for the observed association with SNP rs165934. The associations of *PSEN1* rs165934 and *MAML2* rs76032516 of the Notch signalling pathway genes with OS in Chinese EOC patients are novel findings, which need to be validated in other large and independent studies.

## 1 INTRODUCTION

In the United States, it is estimated that approximately 22 400 patients were diagnosed with epithelial ovarian cancer (EOC) in 2017, while approximately 14 080 women died from this disease.^1^ In China, EOC is also one of the most common cancers, with about 52 100 new cases and about 22 500 deaths estimated in 2015.^2^ While the EOC survival data are not publically available from China,^3^ EOC patients of all stages experienced a poor survival with a 5‐year survival of 46% in the United States as estimated by American Cancer Society.^1^ Therefore, it is essential to identify predictive factors of death risk of EOC patients. In addition to clinical and demographic factors, molecular biomarkers could be promising prognostic or predictive factors for EOC. For example, molecular epidemiological studies have demonstrated that the altered expression levels of *BRCA1* and *BRCA2* as a result of mutations are associated with individual variation of clinical outcomes of EOC patients.^4^, ^5^ The genomewide association study (GWAS) approach has also been used to identify susceptibility loci associated with clinical outcomes in European EOC patients.^6^ Recently, the availability of published GWAS data has promoted the association analysis for risk and outcomes of cancer using a hypothesis‐driven pathway approach.^7^, ^8^

The evolutionarily conserved Notch signalling pathway has been identified to be critical in regulating cell differentiation, proliferation, apoptosis and cell‐cell communication.^9^, ^10^ The Notch signalling pathway consists of four Notch receptors (Notch1‐4) and five ligands (Delta‐like1, Delta‐like3, Delta‐like4, Jagged1 and Jagged2) in mammals.^11^, ^12^, ^13^, ^14^ The initiation of signalling is the combination of Notch ligands with their receptors, which could in turn affect downstream effectors through releasing the intracellular domain of the receptor that is activated by a cascade of proteolytic cleavages mediated by γ‐secretase. For example, it has been reported that NOTCH3 participates in the pathogenesis of EOC recurrence through enhancing carboplatin resistance of cancer cells.^15^ A high *NOTCH4* mRNA expression level was revealed to be significantly correlated with a favourable overall survival (OS) of EOC patients and thus was regarded as a prognostic factor.^16^ As the γ‐secretase inhibitors in the Notch signalling pathway, *DAPT* and *MK‐0752* have been reported to be highly promising therapeutic drug targets for treatment of EOC patients.^17^, ^18^ Recently, a 10‐gene signature of the Notch signalling pathway, including *FZD3*,* HES1*,* PSEN2*,* JAG2*,* PPARG*,* FOS*,* HEY1*,* CDC16*,* MFNG* and *EP300*, has been identified to be associated with a higher risk of recurrence of EOC.^19^ Therefore, the Notch signalling pathway has been suggested to have biological significance and important value in the prognosis of EOC.

The effects of genetic variants in Notch signalling pathway genes on survival of cancer patients have been identified in several cancer types, including cutaneous melanoma,^20^ non‐small‐cell lung cancer 7 and hepatocellular cancer.^21^ In addition, the deregulation of Notch signalling pathway genes is also involved in the development of platinum resistance and recurrence of EOC.^22^ Therefore, we hypothesize that genetic variants in Notch signalling pathway genes contribute to death risk of EOC. To test the hypothesis, we investigated the role of genetic variants of 76 genes in the Notch signalling pathway on OS of EOC patients using available genotyping data from a previously published GWAS data set in a single cancer centre.^23^

## 2 MATERIALS AND METHODS

### 2.1 Study population

The recruitment of participants has been described previously.^23^ In brief, the patients included in this analysis were originally from the Shanghai Ovarian Cancer Study (SOCS), who were consecutively recruited from Fudan University Shanghai Cancer Center (FUSCC) between March 2009 and August 2012. In total, 503 EOC patients with available genotyping and survival data were eligible for this study. All patients were considered sporadic cases who were unrelated ethnic Han Chinese with histologically confirmed EOC.

All of the whole blood samples used for genotyping were obtained from the tissue bank of FUSCC, and the patients had signed informed consent for donating their blood samples to the tissue bank of FUSCC. The study was conducted in accordance with the Declaration of Helsinki and approved by the FUSCC Research Ethics Committee.

### 2.2 Genotyping data and quality control

According to the databases of Molecular Signatures (MsigDB, http://www.broadinstitute.org/gsea/msigdb/search.jsp), 76 Notch signalling pathway genes located on the autosomes with their ±2‐Kb flanking regions (hg19.) were selected for the analysis. As a result, a total of 1740 single nucleotide polymorphisms (SNPs) were genotyped and included in the analysis.

The genotyped data of the GWAS study were generated by the Illumina HumanOmni Zhonghua‐8 BreadChip, and the detailed genotyping information was described previously.^21^ Systematic quality control (QC) was applied to the raw genotyping data before the analysis, and the exclusion criteria of loci were as follows: (i) without mapping to autosomal chromosomes; (ii) with a low call rate in GWAS samples (<95%); (iii) with MAF < 0.05; and (iv) with Hardy‐Weinberg equilibrium *P* < 1 × 10^−5^. After QC, a total of 1273 SNPs within these 75 genes were available from the GWAS genotyping data for the final analysis (Table S1).

### 2.3 False‐positive report probability

False‐positive report probability (FPRP) is the probability of false‐positive association between genetic variants and disease under the given statistically significant findings. In brief, three factors could account for the value of FPRP: the assumed prior probability, an observed *P* value, and statistical power to detect the hazards ratio (HR) of the alternative hypothesis at the given *P* value. For the results of all the selected SNPs, we assigned a prior probability of .01 to detect an HR of 2.0 for an association with genotypes of each SNP. In addition, BFDP (the Bayesian false‐discovery probability) was used with a cut‐off value of 0.75 as recommended,^24^ because BFDP shares the ease of calculation of the recently proposed false‐positive report probability (FPRP) but uses more information, has a noteworthy threshold defined naturally in terms of the costs of false‐discovery and non‐discovery, and has a sound methodological foundation. In a multiple testing situation, BFDP is straightforward to estimate the expected numbers of false discoveries and false non‐discoveries. The difference between FPRP and BFDP is that FPRP uses the observed significance region, whereas BFDP used the q value that has a fixed region, which allows the false‐discovery rate (FDR) to be controlled, a property not inherited by FPRP.^24^ Therefore, we assumed that SNPs with FPRP values <0.2 or BFDP values <0.75 were considered statistically noteworthy for the identified significant associations.

### 2.4 Statistical analysis

The main analysis of the data was to evaluate the associations between genetic variants of 75 genes in the Notch signalling pathway and OS, and the latter was defined as the time interval between the date of histological diagnosis and the date of last follow‐up or time of death. The analysis of OS was conducted for patients who were known to have a minimum of cytoreductive surgery for the first‐line treatment followed by chemotherapy. For the subgroups of patients known to have received standard chemotherapy (≥4 cycles of paclitaxel and platinum at 3‐weekly intervals) after surgery, they were hereafter referred to as having the “standard chemotherapy”; and for the subgroups of patients known to have received chemotherapy without definite cycles or less than 4 cycles, they were hereafter referred to as having the “general chemotherapy.”

The strength of associations between genetic variants and OS of EOC patients were first estimated by computing HRs and their 95% confidence intervals (CIs) by univariate Cox proportional hazards regression analysis. Multivariable Cox regression analysis was then used to evaluate the associations between each genetic variant and OS with adjustment for residue status (nil vs. any cytoreduction), tumour stage (International Federation of Gynecology and Obstetrics [FIGO] stages I‐IV), histological types (serous and others), tumour grade (low vs. high), age at diagnosis and chemotherapy methods (standard vs. general). After the multiple testing corrections, the SNPs identified together with clinical variables were entered into a stepwise Cox analysis model to explore the optimal predictors of OS for EOC patients.

Then, we estimated the effects of risk genotypes on cumulative survival by Kaplan‐Meier curves and log‐rank tests. Time‐dependent receiver operating characteristic (ROC) curve was used to estimate the predictive value of genetic variants combined with clinical variables in both additive and dominant models. Area under the curve (AUC) of ROC curves and concordance index (*C‐*index) were used to illustrate the performance of the model. The combined effects of putative genotypes associated with death risk were also analysed.

Because the SNPs identified from the GWAS data set are tagging SNPs, there is a possibility that the true associated SNPs are obscure. Therefore, the SNPs that were in high linkage disequilibrium (LD) with the tagging SNPs were also identified by using the online tool SNPInfo (https://snpinfo.niehs.nih.gov/) with the 1000 Genomes Project data. To explore potential function of the loci identified from the study, the online tool RegulomeDB (http://www.regulomedb.org/) was used to predict putative function of SNPs identified. We further estimated the correlations between the loci and their corresponding mRNA expression levels of the genes in the ovaries by using the on online tool GTEx database (http://www. gtexportal. org/home/). All statistical analyses were achieved by R (version 3.2.4), SAS (version 9.4; SAS Institute, Cary, NC, USA) software and PLink (version 1.09) (http://pngu. mgh. harvard. edu/purcell/plink/).^25^ All reported *P* values were two‐sided, and *P* < .05 was considered statistically significant.

## 3 RESULTS

### 3.1 Patients’ characteristics and associations with survivals

**Figure 1:**
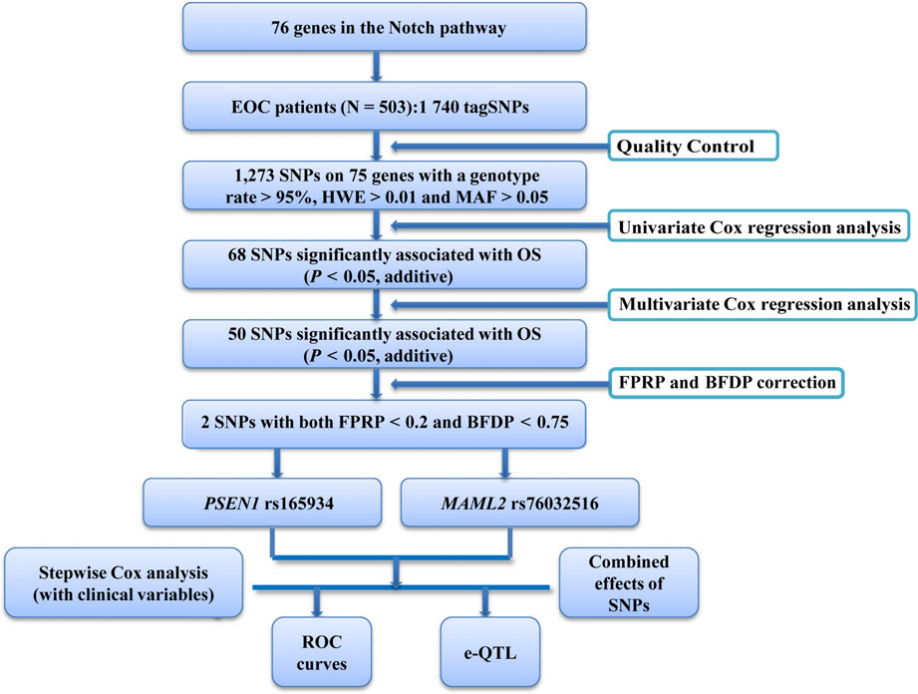
Flow chart of SNP selection in Notch pathway genes and analytic process. EOC, epithelial ovarian cancer; SNP, single nucleotide polymorphisms; HWE, Hardy‐Weinberg equilibrium; MAF, minor allele frequency; FPRP, false‐positive report probability; FDR, false‐discovery rate; eQTL, expression quantitative trait loci

A total of 480 EOC patients were eligible and included in the final analysis after removal of those without survival data. The study analysis flow chart is shown in Figure 1.

The median follow‐up time was 47 months, during which 219 (45.6%) patients died. The EOC patients had an age range between 20 and 81 years (with a mean age of 54.3 ± 10.3 years) at diagnosis. Of all the EOC patients, 328 (68.3%) patients were pathologically diagnosed as serous, and 31 (6.5%) patients were endometrioid, and 20 (4.2%) patients were clear cell. Compared with serous patients, endometrioid patients (endometrioid vs. serous: Crude HR 0.37, 95% CI 0.18‐0.75, *P* = .006; Adjusted HR 0.33, 95% CI 0.16‐0.71, *P* = .005) and clear cell patients (clear cell vs. serous: Crude HR 0.16, 95% CI 0.04‐0.64, *P* = .010; Adjusted HR 0.16, 95% CI 0.04‐0.65, *P* = .011) were associated with a favourable survival by univariate and multivariate Cox regression analysis. Patients were divided as FIGO stage, with 284 (59.2%) being stage III and 69 (14.4%) being stage IV. As expected, both III and IV stage patients showed a poorer survival, with stage I patients as a reference (FIGO III vs. FIGO I: crude HR 7.22, 95% CI 1.79‐29.15, *P* = .006; adjusted HR 6.59, 95% CI 1.61‐26.92, *P* = .009. FIGO IV vs. FIGO I: crude HR 5.61, 95% CI 1.33‐23.68, *P* = .019; adjusted HR 7.60, 95% CI 1.73‐33.38, *P* = .007). Additionally, a total of 306 (63.8%) patients completed standard chemotherapy, while 174 (36.3%) patients received general chemotherapy (chemotherapy without definite cycles or less than 4 cycles) had a higher death risk (General vs. Standard: crude HR 1.63, 95% CI 1.25‐2.13, *P* < .001; adjusted HR 1.79, 95% CI 1.35‐2.35, *P* < .001). The demographic characteristics of patients and their associations with OS are summarized in Table 1.

**Table 1:** Clinical characteristics of EOC patients and their associations with OS

Variables	Patients No. (%)	Death No. (%)	MST (mo)	Crude HR (95% CI)	*P*	Adjusted HR^a^ (95% CI)	*P* a
Age at diagnosis							
<50	138 (28.8)	58 (42.0)	45.0	1.00 (ref.)		1.00 (ref.)	
≥50	342 (71.2)	161 (47.1)	46.0	1.22 (0.90‐1.65)	.194	1.21 (0.89‐1.64)	.219
Grade							
Low	19 (4.0)	7 (36.8)	44.0	1.00 (ref.)		1.00 (ref.)	
High	461 (96.0)	212 (46.0)	45.5	1.27 (0.60‐2.71)	.530	1.10 (0.51‐2.37)	.811
Histology							
Serous	328 (68.3)	160 (48.8)	43.0	1.00 (ref.)		1.00 (ref.)	
Mucinous	18 (3.8)	10 (55.6)	26.5	1.38 (0.72‐2.63)	.332	1.42 (0.68‐2.94)	.348
Endometrioid	31 (6.5)	8 (25.8)	73.0	0.37 (0.18‐0.75)	.006	0.33 (0.16‐0.71)	.005
Clear cell	20 (4.2)	2 (10.0)	64.0	0.16 (0.04‐0.64)	.010	0.16 (0.04‐0.65)	.011
Others	83 (17.3)	39 (47.0)	47.0	0.87 (0.61‐1.23)	.431	0.84 (0.58‐1.20)	.326
FIGO Stage							
I	17 (3.5)	3 (17.6)	76.0	1.00 (ref.)		1.00 (ref.)	
II	62 (12.9)	24 (38.7)	52.5	2.56 (0.75‐8.72)	.132	3.58 (0.94‐13.59)	.061
III	284 (59.2)	147 (51.8)	42.5	7.22 (1.79‐29.15)	.006	6.59 (1.61‐26.92)	.009
IV	69 (14.4)	29 (42.0)	44.0	5.61 (1.33‐23.68)	.019	7.60 (1.73‐33.38)	.007
Unknown^b^	48 (10)	16 (33.3)	56.5	2.35 (0.68‐8.16)	.179	5.56 (0.58‐53.38)	.137
Residue disease^c^							
Nil	241 (50.2)	106 (44.0)	46.0	1.00 (ref.)		1.00 (ref.)	
Any	139 (29.0)	74 (53.2)	31.0	1.51 (1.12‐2.03)	.007	1.26 (0.92‐1.72)	.148
Unknown^b^	100 (20.8)	39 (39.0)	58.0	0.68 (0.46‐0.99)	.042	0.53 (0.33‐0.83)	.005
Chemotherapy^d^							
Standard	306 (63.8)	127 (41.5)	51.0	1.00 (ref.)		1.00 (ref.)	
General	174 (36.3)	92 (52.9)	32.0	1.63 (1.25‐2.13)	<.001	1.79 (1.35‐2.35)	<.001

EOC, epithelial ovarian cancer; OS, overall survival; MST, median survival time; HR, hazard ratio; mo, months; CI, confidence interval.

a Obtained in multivariate Cox regression analysis with adjustment for age at diagnosis, grade, histology, FIGO stage, residue and chemotherapy.

b Unknown refers to the patient without concrete records of the information.

c Residue disease—nil (residue < 1 cm after primary surgery); any (residue ≥ 1 cm).

d Chemotherapy methods: Standard—patients had received standard chemotherapy (≥4 cycles of paclitaxel and platinum at 3‐weekly intervals) after surgery; General—had received chemotherapy without definite cycles or less than 4 cycles.

### 3.2 Cox analysis of associations between SNPs and OS

Initially, we performed univariate Cox regression analysis with an additive model for each of SNPs. A total of 68 SNPs were individually and significantly associated with OS at *P* < .05. Furthermore, when the association of each SNP with OS was assessed by multivariate Cox regression analysis with adjustment for age of diagnosis, tumour grade, FIGO staging, histological types, residue status and chemotherapy, 50 of those 68 SNPs remained significant at *P* < .05 in an additive genetic model (Table S2). Finally, two SNPs (*PSEN1* rs165934 and *MAML2* rs76032516) remained noteworthy with FPRP < 0.2 and BFDP < 0.75 (Table S2), and the identified associations were visualized in a Manhattan plot (Figure S1). However, the limited findings are likely due to the limited sample size and possibly by chance.

**Figure 2:**
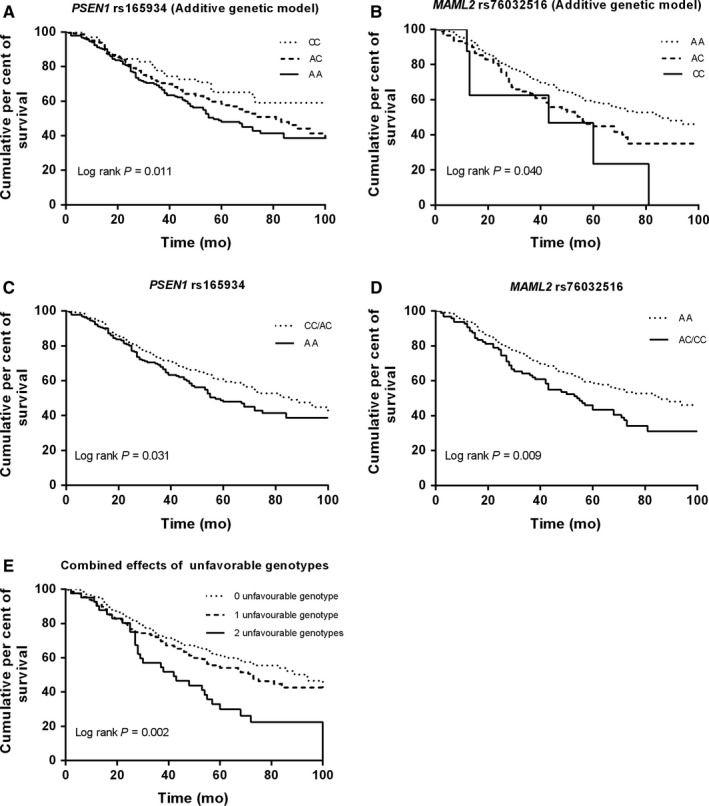
Kaplan‐Meier overall survival curves by genotypes of *PSEN1* rs165934 and *MAML2* rs76032516. A, *PSEN1* rs165934; B, *MAML2* rs76032516; C, *PSEN1* rs165934 unfavourable genotypes (AA vs. AA/AC); D, *MAML2* rs76032516 unfavourable genotypes (AC/CC vs. AA); E, combined risk of unfavourable genotypes (*PSEN1* rs165934 AA and *MAML2* rs76032516 AC/CC)

Specifically, in the multivariable analysis (Table 2), we found that the *PSEN1* rs165934 AA genotype was associated with a poor survival, compared with the CC (adjusted HR = 1.92, 95% CI = 1.20‐3.06 and *P* = .006) and CC + CA genotypes (adjusted HR = 1.40, 95% CI = 1.07‐1.84, and *P* = .014). Besides, the *MAML2* rs76032516 was also associated with a poor survival: adjusted HR = 1.50, 95% CI = 1.09‐2.06 and *P* = .013 for the comparison of AC vs. AA; adjusted HR = 3.01, 95% CI = 1.31‐6.91 and *P* = .009 for the comparison of CC vs. AA, respectively; and adjusted HR = 1.58, 95% CI = 1.16‐2.14, *P* = .004 for *MAML2* rs76032516 AC + CC genotypes, compared with the AA genotype. When we combined these two loci to estimate their joint effects on OS, patients carried two unfavourable genotypes (ie the *PSEN1* rs165934 AA and *MAML2* rs76032516 AC/CC) had a higher death risk (HR = 2.34, 95% CI = 1.53‐3.59, *P* < .001) than those without unfavourable genotype; patients carried one unfavourable genotype also had a higher death risk with a borderline significance (HR = 1.32, 95% CI = 0.99‐1.77, *P* = .056) than those without unfavourable genotype; and the trend test was statistically significant (*P* < .001). The classification performance of risk genotypes of *PSEN1* rs165934 (Figure 2A,C) and *MAML2* rs76032516 (Figure 2B,D), and their combined effects (Figure 2E) on OS was illustrated by Kaplan‐Meier curves.

**Table 2:** Univariate and multivariate Cox analysis of associations between SNPs and OS in EOC patients

Genotypes	Patients No. (%)	Deaths No. (%)	MST (mo)	Crude HR (95% CI)	*P*	Adjusted HR^a^ (95% CI)	*P* a
*PSEN1* rs165934							
CC	64 (13.3)	22 (10.0)	60.0	1.00 (ref.)		1.00 (ref.)	
AC	229 (47.7)	102 (46.6)	45.0	1.37 (0.88‐2.14)	.169	1.39 (0.89‐2.19)	.151
AA	187 (39.0)	95 (43.4)	43.0	1.76 (1.11‐2.78)	**.015**	1.92 (1.20‐3.06)	**.006**
Trend test					**.012**		**.004**
CC	64 (13.3)	22 (10.0)	60.0	1.00 (ref.)		1.00 (ref.)	
AC+AA^c^	416 (86.7)	197 (90.0)	44.0	1.53 (1.00‐2.34)	**.051**	1.60 (1.04‐2.45)	**.033**
CC+AC	293 (51.0)	124 (56.6)	49.0	1.00 (ref.)		1.00 (ref.)	
AA^d^	187 (39.0)	95 (43.4)	43.0	1.34 (1.02‐1.75)	**.033**	1.40 (1.07‐1.84)	**.014**
*MAML2* rs76032516							
AA	383 (79.8)	159 (72.6)	46.0	1.00 (ref.)		1.00 (ref.)	
AC	89 (18.5)	54 (24.7)	43.0	1.41 (1.03‐1.93)	**.030**	1.50 (1.09‐2.06)	**.013**
CC	8 (1.7)	6 (2.7)	29.0	2.63 (1.16‐5.94)	**.021**	3.01 (1.31‐6.91)	**.009**
Trend test					**.004**		**.001**
AA	383 (79.8)	159 (72.6)	46.0	1.00 (ref.)		1.00 (ref.)	
AC+CC^c^	97 (20.2)	60 (27.4)	43.0	1.48 (1.10‐2.00)	**.010**	1.58 (1.16‐2.14)	**.004**
AA+AC	472 (98.3)	213 (97.3)	45.5	1.00 (ref.)		1.00 (ref.)	
CC^d^	8 (1.7)	6 (2.7)	29.0	2.40 (1.06‐5.40)	**.035**	2.74 (1.20‐6.22)	**.017**
Combined effects of unfavourable genotypes^b^							
0	238 (49.6)	95 (43.4)	49.0	1.00 (ref.)		1.00 (ref.)	
1	201 (41.9)	94 (42.9)	44.0	1.24 (0.94‐1.66)	.134	1.32 (0.99‐1.77)	.056
2	41 (8.5)	30 (13.7)	38.0	2.13 (1.41‐3.22)	<**.001**	2.34 (1.53‐3.59)	<**.001**
Trend test					**.001**		<**.001**
0‐1	439 (91.5)	189 (86.3)	46.0	1.00 (ref.)		1.00 (ref.)	
2	41 (8.5)	30 (13.7)	38.0	1.89 (1.28‐2.79)	**.001**	2.04 (1.37‐3.03)	**.001**

SNP, single nucleotide polymorphisms; OS, overall survival; EOC, epithelial ovarian cancer; MST, median survival time; HR, hazard ratio; mo, months; CI, confidence interval.

The results were in bold, if *P* < .05.

a The multivariate Cox analysis was adjusted for age at diagnosis, tumour grade, FIGO stage, histological types, residual disease and chemotherapy.

b Unfavourable genotypes: *PSEN1* rs165934 AA; *MAML2* rs76032516 AC/CC.

c The analysis was in a dominant model.

d The analysis was in a recessive model.

### 3.3 Survival analysis of SNPs and OS

**Figure 3:**
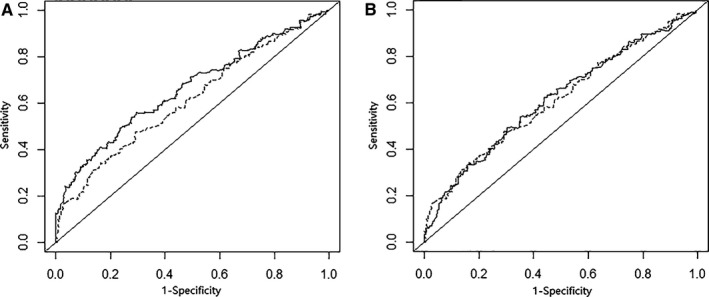
Survival ROC curves for prediction of overall survival based on clinical variables, and the combined effect of risk genotypes with calculating in an additive model (A) and in a dominant model (B)

In addition, we constructed time‐dependent ROC curves to identify whether survival‐associated SNPs (*PSEN1* rs165934 and *MAML2* rs76032516) in the presence of clinical variables (ie. age at diagnosis, tumour grade, histology, FIGO stage, residue disease and chemotherapy methods) could better discriminate survival of patients. Compared with the survival prediction model with clinical variables alone, the model with addition of the two identified SNPs significantly improved the capability of discriminating survival (AUC, 0.662 vs. 0.612 in an additive genetic model and; AUC0.662 vs. 0.643 in a dominant genetic model). Moreover, the combined analysis indicated a higher *C*‐index for the survival prediction model than analysis of considering clinical variables alone, in either an additive (combined vs. clinical variables alone, additive model, *C* ‐index: 0.647 vs. 0.619, *P* < .001) or a dominant genetic model (*C* ‐index: 0.636 vs. 0.619, *P* < .001) (Figure 3).

Furthermore, we performed the stepwise multivariate Cox regression analysis to select the optimal predictors of OS in EOC patients, with the clinical variables listed in Table 1 and the two SNPs identified (*PSEN1* rs165934 and *MAML2* rs76032516). In addition to age at diagnosis (HR = 1.02, 95% CI 1.01‐1.03), chemotherapy (HR = 1.82, 95% CI 1.38‐2.40), histology (HR = 0.89, 95% CI 0.81‐0.98), the two SNPs, *PSEN1* rs165934 (HR = 1.36, 95% CI 1.11‐1.67) and *MAML2* rs76032516 (HR = 1.54, 95% CI 1.17‐2.02) were also predictors of OS for EOC patients in an additive model (Table 3).

**Table 3:** Stepwise multivariate Cox analysis for predicting OS in EOC patients

Variables	Comparison	HR^a^	95% CI	*P*
Age at diagnosis	In years	1.02	1.01‐1.03	.007
Chemotherapy	Yes vs. No	1.82	1.38‐2.40	<.001
Histology	Serous vs. others	0.89	0.81‐0.98	.013
*PSEN1* rs165934^b^	A vs. C	1.36	1.11‐1.67	.003
*MAML2* rs76032516^b^	C vs. A	1.54	1.17‐2.02	.002

OS, overall survival; EOC, epithelial ovarian cancer; HR, hazard ratio; CI, confidence interval.

a Obtained in a stepwise multivariate Cox regression analysis, and variables including age, tumour grade, histological type, FIGO stage, residue disease, chemotherapy and the two SNPs identified (*PSEN1* rs165934, *MAML2* rs76032516).

b Obtained in an additive model.

### 3.4 Stratified analysis between unfavourable genotypes and EOC survival

We performed stratified analysis to assess differential effects of clinical variables on death risk associated with unfavourable genotype groups (Table 4). Overall, patients carrying two unfavourable genotypes tended to have an evidently increased death risk in subgroups of patients ≥50 year at diagnosis, high tumour grade, serous EOC, FIGO stage III‐IV, residue disease as well as both subgroups of chemotherapies, compared with that of patients carrying 0‐1 unfavourable genotype (*P* < 0.05 for all).

**Table 4:** Stratification analysis for associations between combined risk of unfavourable SNPs and OS of EOC patients

Stratification variables	0‐1 unfavourable genotypes		2 unfavourable genotypes		HR (95% CI)	*P*	*P* a
	Patients No. (%)	Deaths No. (%)	Patients No. (%)	Deaths No. (%)			
Age at diagnosis							
<50	129 (26.9)	53 (24.2)	9 (1.9)	5 (2.3)	1.39 (0.55‐3.49)	.486	.315
≥50	310 (64.6)	136 (62.1)	32 (6.7)	25 (11.4)	2.01 (1.31‐3.09)	**.002**	
Grade							
Low	16 (3.3)	6 (2.7)	3 (0.6)	1 (0.5)	0.82 (0.10‐6.87)	.858	.249
High	423 (88.1)	183 (83.6)	38 (7.9)	29 (13.2)	1.99 (1.34‐2.95)	**.001**	
Histological types							
Serous	301 (62.7)	138 (63.0)	27 (5.6)	22 (10.0)	1.91 (1.21‐3.01)	**.005**	.721
Others^b^	138 (28.8)	51 (23.3)	14 (2.9)	8 (3.7)	2.07 (0.97‐4.40)	.059	
FIGO Stage							
I‐II	67 (14.0))	21 (9.6)	12 (2.5)	6 (2.7)	1.61 (0.63‐4.10)	.318	.095
III‐IV	327 (68.1)	155 (70.8)	26 (5.4)	21 (9.6)	1.97 (1.24‐3.11)	**.004**	
Unknown^c^	45 (9.4)	13 (5.9)	3 (0.6)	3 (1.4)	8.53 (2.31‐31.42)	**.001**	
Residue disease^a^							
Nil	218 (45.4)	93 (42.5)	23 (4.8)	13 (5.9)	1.12 (0.61‐2.04)	.717	.542
Any	129 (26.9)	64 (29.2)	10 (2.1)	10 (4.6)	2.96 (1.50‐5.86)	**.002**	
Unknown	92 (19.2)	32 (14.6)	8 (1.7)	7 (3.2)	5.30 (2.27‐12.39)	<**.001**	
Chemotherapy methods^d^							
Standard	276 (57.5)	106 (48.4)	30 (6.3)	21 (9.6)	1.92 (1.20‐3.09)	**.007**	.190
General	163 (34.0)	83 (37.9)	11 (2.3)	9 (4.1)	2.42 (1.21‐4.84)	**.013**	

The results were in bold, if *P* < .05.

SNP, single nucleotide polymorphisms; OS, overall survival; EOC, epithelial ovarian cancer; HR, hazard ratio; CI, confidence interval.

a *P*, chi‐square test for genotype distribution between the two groups; Unfavourable genotypes: *PSEN1* rs165934 AA; *MAML2* rs76032516 AC/CC.

b Histology—others (combined mucinous, endometrioid, clear cell and others types of EOC).

c Unkown‐ the patient without concrete records of the information;

d Chemotherapy methods‐Standard ‐patients have received standard chemotherapy (>=4 cycles of paclitaxel and platinum at 3‐weekly intervals) after surgery; General—have received chemotherapy without definite cycles or less than 4 cycle.

### 3.5 Correlations between the loci identified and mRNA expression levels

From the online SNPinfo website with genotyping data for Chinese Han Beijing (CHB) subjects, three loci were in high LD with *PSEN1* rs165934 (for rs165932 with *r* ^2^ = .946; rs165935 with *r* ^2^ = .973; and rs177415 with *r* ^2^ = .945) (Figure S2A). We further searched for the functional relevance in the RegulomDB database for *PSEN1* rs165934 and *MAML2* rs76032516, as well as the three high LD loci (rs165932, rs165935 and rs177415), and found that *PSEN1* rs165932, *PSEN1* rs165935, *PSEN1* rs1177415 and *MAML2* rs76032516 were all putatively functional.

**Figure 4:**
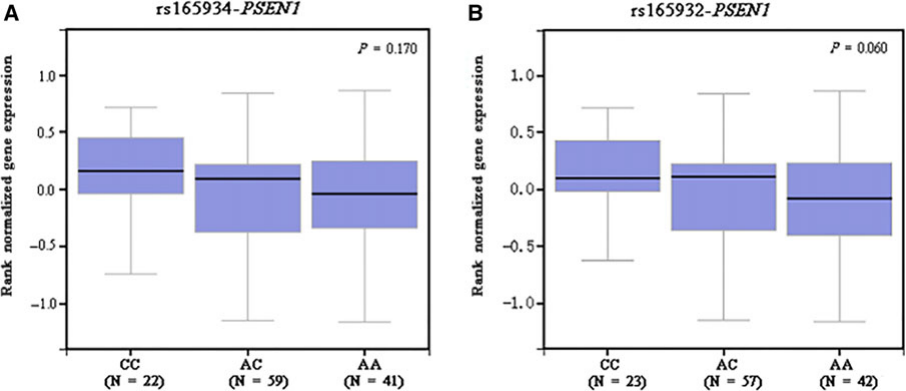
The mRNA expression levels by *PSEN1* rs165934 (A) and rs165932 (B) in ovaries from the expression quantitative trait loci analysis from the GTEx database

To examine the genotypic effect of the four SNPs on their mRNA expression levels, the eQTL analysis was performed using publically available GTEx databases. In the ovarian tissues, there were 22 CC, 59 AC and 41 AA carriers for *PSEN1* rs165934, and the change of A to C showed an increased trend of expression levels of *PSEN1* (*P* = .170) (Figure 4A). We further explored the genetic expression correlated with the change of rs165932 A to C (in high LD with rs165934, *r* ^2^ = .946) in the ovarian tissues (23 CC, 57AC and 42 AA carriers), and we found that *PSEN1* expression levels increased with a borderline significant trend (*P* = .060) (Figure 4B). In addition, the variant of *PSEN1* rs165932 had an effect on OS (crude HR = 0.76, 95% CI = 0.62‐0.93 and *P* = .006; adjusted HR = 0.74, 95% CI = 0.60‐0.90 and *P* = .003), but these HRs were no longer significant after the correction of FPRP < 0.2 (Table S2). Taken together, it is biologically plausible that the associations between *PSEN1* rs165934 A allele (in LD with) and survival might be explained by mRNA expression that is regulated by the rs165932 variant locus. However, the difference in *MAML2* expression levels among the three genotypes for the locus rs76032516 was not statistically evident, which might be because of the limited sample sizes (Figure S2B).

## 4 DISCUSSION

To our knowledge, this is the first study to evaluate the associations of genetic variants of Notch pathway genes with OS in Han Chinese EOC patients. We found that *PSEN1* rs165934 A > C and *MAML2* rs76032516 A > C were associated with survival of patients, and the underlying mechanism of the *PSEN1* rs165934 A > C on survival is likely to result from expression regulation by the change of rs165932 A to C that within the same LD block. Therefore, our results suggest that the observed effects of *PSEN1* rs165934 in Notch signalling pathway on survival of EOC patients are biologically plausible. However, the possible mechanism of the *MAML2* rs76032516 A > C on the survival remains to be unravelled.

Notch signalling is an evolutionarily conserved mechanism to control cells’ response to intrinsic or extrinsic developmental cues that are obligatory to unfold specific developmental programs.^9^ The Notch activity involves in the process of differentiation, proliferation, and apoptotic programs in all stages of development of the organisms. There is evidence that aberrant activation of the Notch signalling pathway plays a crucial role in the process of ovarian carcinogenesis and chemoresistance in ovarian cancer patients.^26^, ^27^ A previous GWAS‐based pathway analysis found that *NCOR2*,* NCSTN* and *MAML2* variants in the Notch signalling pathway predicted survival in patients with cutaneous melanoma.^20^ Besides, variants in *ADAM12* and *TLE1* were also found to be associated with a poor survival in non‐small‐cell lung cancer patients.^7^ In the present gene‐set analysis using the available GWAS data set in Han Chinese women, we observed that variants in *PSEN1* and *MAML2* were associated with OS of EOC patients.

The OS‐associated SNP *PSEN1* rs165934 is located in the intron between the exons 8 and 9. The present study showed that carriers of the *PSEN1* rs165934 A allele had a poorer OS than C allele carriers and that there was a non‐significant trend of decreased *PSEN1* mRNA expression levels in ovarian tissues due to the rs165934 C to A change. Nevertheless, rs165932 in LD with rs165934 (*r* ^2^ = .946) in a LD block may be responsible for regulation of the gene expression. Collectively, the associations between rs165932 C allele with high tumour tissue levels of *PSEN1* mRNA may have led to a better survival. However, the expression data were derived from European populations whose distribution of the genotypes is likely to be different from that of Chinese populations. Therefore, additional studies on mRNA expression levels by *PSEN1* rs165932 genotypes among Chinese patients are needed to validate our findings.

PSEN1 (Presenilin‐1) is a catalytic component of the γ‐secretase complex that is responsible for the cleavage of the transmembrane protein, which in turn induces the release of the Notch intracellular domain (NICD). The transportation of NICD to the nucleus could activate transcription of target genes in conjunction with the suppressor of hairless and mastermind proteins.^28^, ^29^ PSEN1 is a ubiquitously expressed multi‐transmembrane domain protein, primarily located on the endoplasmic reticulum.^30^ The roles of PSEN1 have been demonstrated to be involved in various tumorigenic processes, such as cell proliferation, apoptosis and cell adhesion.^31^ A previous study found that PSEN1 was involved in the process of invasion and metastasis of gastric cancer both *in vitro* and *vivo,* 30 which may be regarded as a potential biomarker and therapeutic target. On the other hand, PSEN1 was also reported to be a tumour suppressor, and the enhanced expression of *PSEN1* was associated with a favourable disease‐free survival for patients with breast cancer^32^ and skin cancer.^33^ However, little is known for its role in ovarian cancer. Although *PSEN1* rs165934 has no direct role in regulating the gene expression at the transcription level, it is located in the DNase I hypersensitive site based on the ENCODE project database from University of California Santa Cruz (UCSC). DNase I hypersensitive sites, representing open and active chromatins, are accessible to regulatory proteins and marks *cis*‐regulatory enriched regions. The variant is shown to be located in an H3k27Ac enrichment region according to the ChIP‐seq data from ENCODE (The Encyclopedia of DNA Elements). Therefore, the change of rs165934 A to C could have an effect on the gene's function on regulating the expression of PSEN1 and thus could affect patients’ survival. These putative novel findings give us a hint that *PSEN1* could be a potential molecular biomarker for survival of EOC patients, once validated. Further functional studies are needed to reveal the underlying biological mechanisms of *PSEN1* on survival.

*MAML2* (mastermind‐like transcriptional coactivator 2) rs76032516 is located in the third intron of the gene on 11q21, which is likely to be functional as predicted by RegulomeDB. The MAML2 protein contains a conserved basic domain that could bind to the ankyrin repeat domain of the intracellular domain of the Notch receptors (ICN1‐4) in their N‐terminus. The protein binds to an extended groove that is formed by the interaction of CBF1, suppressor of Hairless, LAG‐1 with ICN, which could positively regulate the Notch signalling pathway.^34^, ^35^ In fact, the translocation of *MAML2* could create a fusion oncogene *MECT1*/*MAML2*, which could be involved in the process of disrupting the normal cell cycle, differentiation and tumour development, exerting an oncogenic role in mucoepidermoid carcinoma.^36^ Recently, the oncogenic *CRCT3*‐*MAML2* fusion was also identified to be associated with a better survival of patients with mucoepidermoid carcinoma.^37^ Although *MAML2* rs76032516 was found to be significantly associated with OS of EOC patients in the present study, the mechanism of *MAML2* underlying the effect on the survival was not identified in the present study, nor has not been reported in the literature. Thus, our findings need to be further validated for its functional relevance through in vivo and in vitro studies of *MAML2* variants and the gene in the future.

However, the present study has several limitations. First, an inherent limitation was the observational design of the study with limited information on available clinical outcomes, such as chemotherapy‐related outcomes, progress free survival (PFS) and objective response rate; second, we were not able to explore the exact mechanism of the loci that were identified to have an effect on survival of EOC; third, we did not have the mutation profile of the tumours that may have impact on survival; fourth, we did not have an access to suitable and comparable target tissue samples from the subjects included in the present study to explore the possible molecular mechanism underlying the observed associations; and finally, the results of the present study may be a chance finding by a limited sample size without additional validation. Therefore, further large and independent studies are required to validate our findings to reduce the positive results by chance.

## CONFLICT OF INTEREST

The authors confirm that there are no conflict of interests.
