# Supplementary figures and images for: Variants in Notch signalling pathway genes, PSEN1 and MAML2, predict overall survival in Chinese patients with epithelial ovarian cancer
Source: J Cell Mol Med. 2018 Jul 28;22(10):4975–84. doi: 10.1111/jcmm.13764 (PMC6156353; doi:10.1111/jcmm.13764)

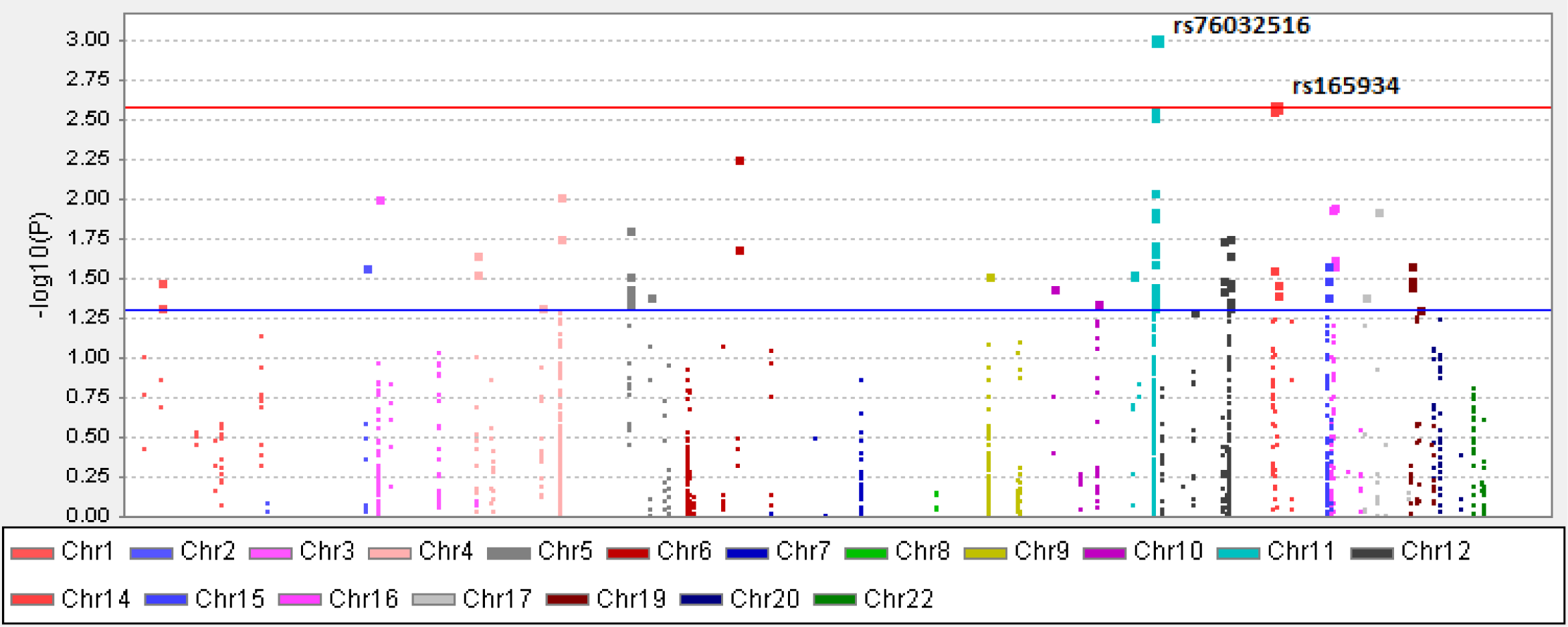

Supplement: Supplementary file 1 [file JCMM-22-4975-s001.tif]

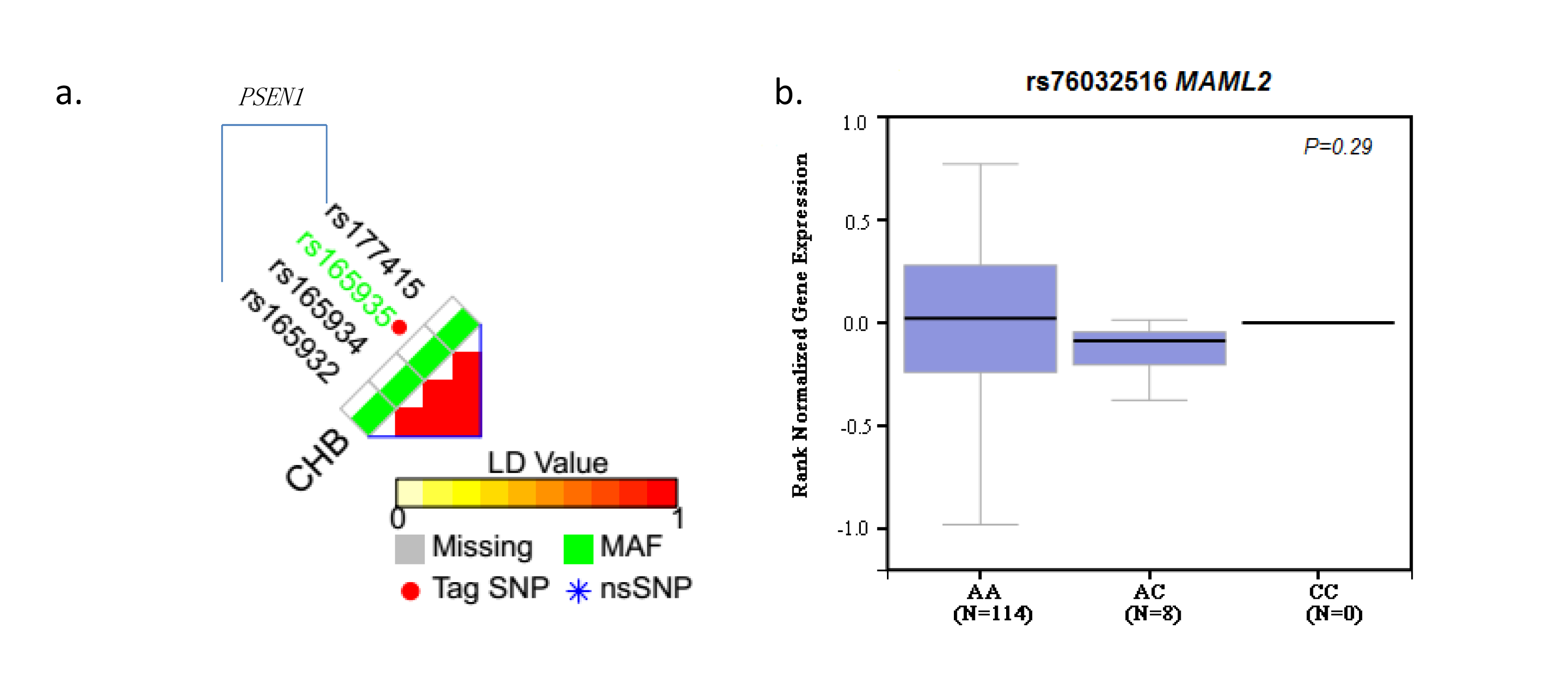

Supplement: Supplementary file 2 [file JCMM-22-4975-s002.tif]
