# Supplementary material for: Variants in Notch signalling pathway genes, PSEN1 and MAML2, predict overall survival in Chinese patients with epithelial ovarian cancer
Source: J Cell Mol Med. 2018 Jul 28;22(10):4975–84. doi: 10.1111/jcmm.13764 (PMC6156353; doi:10.1111/jcmm.13764)
Supplement: Supplementary file 3 [file JCMM-22-4975-s003.docx]

| Table S1. List of genotyped SNPs in the Notch pathway genes | | |
| --- | --- | --- |
| Gene | **Chromosome** | **No. of SNPs included for the analysis** |
| *APH1A* | 1 | 3 |
| *HDAC1* | 1 | 3 |
| *JUN* | 1 | 4 |
| *NCSTN* | 1 | 10 |
| *NOTCH2* | 1 | 6 |
| *PSEN2* | 1 | 11 |
| *CIR1* | 2 | 10 |
| *ADAM17* | 2 | 5 |
| *CTNNB1* | 3 | 6 |
| *GSK3B* | 3 | 19 |
| *DTX3L* | 3 | 8 |
| *DVL3* | 3 | 2 |
| *KAT2B* | 3 | 37 |
| *MAML3* | 4 | 150 |
| *CTBP1* | 4 | 15 |
| *DKK2* | 4 | 15 |
| *RBPJ* | 4 | 12 |
| *APC* | 5 | 20 |
| *CSNK1A1* | 5 | 8 |
| *FBXW11* | 5 | 8 |
| *MAML1* | 5 | 9 |
| *DLL1* | 6 | 6 |
| *MAP3K7* | 6 | 8 |
| *NOTCH4* | 6 | 57 |
| *PPARD* | 6 | 21 |
| *PPP2R5D* | 6 | 7 |
| *HDAC2* | 6 | 4 |
| *FZD1* | 7 | 1 |
| *SSPO* | 7 | 25 |
| *LFNG* | 7 | 1 |
| *FZD1* | 7 | 1 |
| *MYC* | 8 | 4 |
| *NOTCH1* | 9 | 12 |
| *TLE1* | 9 | 66 |
| *CTBP2* | 10 | 18 |
| *DKK1* | 10 | 3 |
| *FRAT1* | 10 | 5 |
| *MAML2* | 11 | 196 |
| *CCND1* | 11 | 2 |
| *DTX4* | 11 | 7 |
| *NCOR2* | 12 | 106 |
| *DTX1* | 12 | 15 |
| *HNF1A* | 12 | 16 |
| *LRP6* | 12 | 19 |
| *WIF1* | 12 | 12 |
| *WNT1* | 12 | 1 |
| *NUMB* | 14 | 19 |
| *SNW1* | 14 | 5 |
| *FOS* | 14 | 9 |
| *JAG2* | 14 | 3 |
| *PSEN1* | 14 | 9 |
| *ADAM10* | 15 | 25 |
| *APH1B* | 15 | 11 |
| *NEDD4* | 15 | 23 |
| *CREBBP* | 16 | 19 |
| *AXIN1* | 16 | 26 |
| *KREMEN2* | 16 | 2 |
| *MAPK3* | 16 | 1 |
| *NKD1* | 16 | 6 |
| *DVL2* | 17 | 5 |
| *NLK* | 17 | 11 |
| *KAT2A* | 17 | 2 |
| *RFNG* | 17 | 2 |
| *AES* | 19 | 11 |
| *NOTCH3* | 19 | 11 |
| *PSENEN* | 19 | 1 |
| *DLL3* | 19 | 5 |
| *NUMBL* | 19 | 5 |
| *RBPJL* | 20 | 3 |
| *CSNK2A1* | 20 | 12 |
| *JAG1* | 20 | 23 |
| *MAPK1* | 22 | 24 |
| *MFNG* | 22 | 5 |
| *TAB1* | 22 | 3 |
| *EP300* | 22 | 18 |
| Total |  | 1,273 |

| Table S2. List of 50 common SNPs in the Notch signaling pathway genes found to be associated with overall survival | | | | | | | | |
| --- | --- | --- | --- | --- | --- | --- | --- | --- |
|  |  |  | **Univariate Cox analysis** | | **Multivariate Cox analysis** | |  |  |
| SNP | **Gene** | **Chromosome** | **HR (95% CI)** | ***P* value** | **HR (95% CI)** | ***P* value** | **FPRP** | **BFDP** |
| rs76032516 | *MAML2* | 11 | 1.48 (1.14-1.92) | 0.004 | 1.58 (1.20-2.07) | **0.001** | **0.088** | 0.651 |
| rs165934 | *PSEN1* | 14 | 0.76 (0.62-0.93) | 0.007 | 0.73 (0.60-0.90) | **0.003** | **0.199** | 0.651 |
| rs17806593 | *MAML2* | 11 | 1.48 (1.09-1.85) | 0.010 | 1.50 (1.15-1.96) | 0.003 | 0.234 | 0.651 |
| rs165932 | *PSEN1* | 14 | 0.76 (0.62-0.93) | 0.006 | 0.74 (0.60-0.90) | 0.003 | 0.213 | 0.651 |
| rs4603268 | *MAML2* | 11 | 1.33 (1.07-1.65) | 0.011 | 1.39 (1.12-1.72) | 0.003 | 0.217 | 0.651 |
| rs12286938 | *MAML2* | 11 | 1.36 (1.05-1.77) | 0.020 | 1.49 (1.14-1.93) | 0.003 | 0.232 | 0.651 |
| rs2499618 | *HDAC2* | 6 | 0.75 (0.61-0.91) | 0.003 | 0.76 (0.62-0.92) | 0.005 | 0.352 | 0.799 |
| rs483333 | *MAML2* | 11 | 0.73 (0.59-0.92) | 0.007 | 0.74 (0.59-0.93) | 0.009 | 0.481 | 0.799 |
| rs2048752 | *NLK* | 17 | 1.49 (1.08-2.07) | 0.016 | 1.55 (1.11-2.15) | 0.009 | 0.646 | 0.799 |
| rs118044954 | *KAT2B* | 3 | 0.62 (0.42-0.91) | 0.014 | 0.60 (0.41-0.89) | 0.010 | 0.583 | 0.799 |
| rs11644593 | *CREBBP* | 16 | 1.36 (1.10-1.67) | 0.005 | 1.32 (1.07-1.63) | 0.011 | 0.523 | 0.799 |
| rs758033 | *AXIN1* | 16 | 1.54 (1.10-2.16) | 0.012 | 1.54 (1.10-2.15) | 0.011 | 0.567 | 0.799 |
| rs17792426 | *NLK* | 17 | 1.47 (1.08-1.99) | 0.015 | 1.49 (1.09-2.02) | 0.012 | 0.537 | 0.799 |
| rs528098 | *MAML2* | 11 | 1.35 (1.07-1.69) | 0.010 | 1.34 (1.07-1.69) | 0.012 | 0.552 | 0.799 |
| rs454886 | *APC* | 5 | 1.29 (1.05-1.58) | 0.014 | 1.28 (1.05-1.57) | 0.015 | 0.609 | 0.799 |
| rs12642915 | *MAML3* | 4 | 1.56 (1.05-2.32) | 0.027 | 1.63 (1.09-2.44) | 0.017 | 0.703 | 0.799 |
| rs73427738 | *DTX1* | 12 | 0.80 (0.66-0.96) | 0.017 | 0.80 (0.66-0.96) | 0.018 | 0.640 | 0.799 |
| rs548175 | *MAML2* | 11 | 1.26 (1.01-1.59) | 0.043 | 1.31 (1.05-1.64) | 0.019 | 0.652 | 0.799 |
| rs6568819 | *HDAC2* | 6 | 1.28 (1.03-1.58) | 0.024 | 1.29 (1.04-1.61) | 0.020 | 0.670 | 0.799 |
| rs2164182 | *MAML2* | 11 | 1.26 (1.01-1.59) | 0.043 | 1.30 (1.04-1.64) | 0.021 | 0.683 | 0.799 |
| rs1250116 | *CTBP1* | 4 | 1.29 (1.02-1.62) | 0.032 | 1.31 (1.04-1.64) | 0.022 | 0.684 | 0.799 |
| rs884421 | *CTBP1* | 4 | 0.78 (0.62-0.98) | 0.032 | 0.77 (0.61-0.96) | 0.022 | 0.684 | 0.799 |
| rs2239316 | *CREBBP* | 16 | 1.24 (1.02-1.50) | 0.029 | 1.25 (1.03-1.52) | 0.023 | 0.697 | 0.799 |
| rs12365545 | *RBPJL* | 21 | 1.27 (1.02-1.58) | 0.034 | 1.29 (1.03-1.60) | 0.025 | 0.715 | 0.799 |
| rs2277741 | *AES* | 19 | 1.42 (1.07-1.89) | 0.017 | 1.39 (1.04-1.86) | 0.026 | 0.727 | 0.799 |
| rs130005 | *CREBBP* | 16 | 0.77 (0.63-0.94) | 0.011 | 0.79 (0.64-0.97) | 0.026 | 0.721 | 0.799 |
| rs17408630 | *PSEN1* | 14 | 1.23 (1.01-1.49) | 0.045 | 1.26 (1.03-1.54) | 0.027 | 0.731 | 0.799 |
| rs1265923 | *CTBP1* | 4 | 0.79 (0.63-1.00) | 0.045 | 0.78 (0.62-0.97) | 0.029 | 0.740 | 0.799 |
| rs77377561 | *APC* | 5 | 1.25 (1.01-1.54) | 0.042 | 1.27 (1.02-1.57) | 0.030 | 0.758 | 0.799 |
| rs383902 | *ADAM10* | 15 | 1.35 (1.04-1.75) | 0.024 | 1.33 (1.03-1.73) | 0.032 | 0.766 | 0.799 |
| rs1732803 | *DTX1* | 12 | 1.25 (1.03-1.51) | 0.025 | 1.23 (1.02-1.50) | 0.032 | 0.762 | 0.799 |
| rs425485 | *AES* | 19 | 0.75 (0.60-0.93) | 0.010 | 0.78 (0.62-0.98) | 0.032 | 0.762 | 0.799 |
| rs11610540 | *NCOR2* | 12 | 0.69 (0.49-0.98) | 0.039 | 0.68 (0.48-0.97) | 0.033 | 0.784 | 0.799 |
| rs4806901 | *AES* | 19 | 0.72 (0.55-0.95) | 0.022 | 0.74 (0.56-0.98) | 0.035 | 0.777 | 0.799 |
| rs4807391 | *AES* | 19 | 1.39 (1.05-1.85) | 0.022 | 1.36 (1.02-1.82) | 0.035 | 0.801 | 0.799 |
| rs10765795 | *MAML2* | 11 | 1.34 (1.02-1.76) | 0.033 | 1.35 (1.02-1.79) | 0.035 | 0.782 | 0.799 |
| rs10831503 | *MAML2* | 11 | 0.81 (0.66-0.99) | 0.036 | 0.81 (0.66-0.99) | 0.036 | 0.779 | 0.799 |
| rs42427 | *APC* | 5 | 0.75 (0.58-0.97) | 0.030 | 0.76 (0.59-0.98) | 0.036 | 0.782 | 0.799 |
| rs465899 | *APC* | 5 | 0.75 (0.58-0.97) | 0.030 | 0.76 (0.59-0.98) | 0.036 | 0.784 | 0.799 |
| rs11001553 | *DKK1* | 10 | 0.69 (0.52-0.92) | 0.010 | 0.74 (0.55-0.98) | 0.036 | 0.788 | 0.799 |
| rs2701623 | *DTX1* | 12 | 1.28 (1.04-1.56) | 0.020 | 1.24 (1.01-1.52) | 0.037 | 0.784 | 0.799 |
| rs479244 | *MAML2* | 11 | 1.23 (1.02-1.48) | 0.028 | 1.22 (1.01-1.47) | 0.038 | 0.792 | 0.799 |
| rs2074222 | *DVL2* | 17 | 0.81 (0.67-1.00) | 0.046 | 0.81 (0.66-0.99) | 0.040 | 0.799 | 0.799 |
| rs2125130 | *CSNK1A1* | 5 | 0.75 (0.59-0.95) | 0.018 | 0.78 (0.61-0.99) | 0.040 | 0.800 | 0.799 |
| rs866006 | *APC* | 5 | 1.32 (1.02-1.70) | 0.037 | 1.31 (1.01-1.70) | 0.041 | 0.803 | 0.799 |
| rs7923776 | *CTBP2* | 10 | 0.81 (0.65-1.00) | 0.044 | 0.81 (0.66-1.00) | 0.044 | 0.815 | 0.799 |
| rs464708 | *APC* | 5 | 1.31 (1.01-1.70) | 0.039 | 1.30 (1.01-1.69) | 0.046 | 0.825 | 0.799 |
| rs2545162 | *APC* | 5 | 0.76 (0.59-0.99) | 0.039 | 0.77 (0.59-1.00) | 0.046 | 0.819 | 0.799 |
| rs10831494 | *MAML2* | 11 | 0.76 (0.61-0.96) | 0.019 | 0.80 (0.64-1.00) | 0.048 | 0.825 | 0.799 |
| rs351771 | *APC* | 5 | 0.76 (0.58-0.98) | 0.033 | 0.76 (0.59-0.97) | 0.039 | 0.817 | 0.799 |
| Abbreviations: HR, hazards ratio; FPRP, false positive report probability (cut-off<0.2); BFDP, Bayesian false-discovery probability (cut-off<0.75). | | | | | | | | |
